# Summertime sea-ice prediction in the Weddell Sea improved by sea-ice thickness initialization

**DOI:** 10.1038/s41598-021-91042-4

**Published:** 2021-06-09

**Authors:** Yushi Morioka, Doroteaciro Iovino, Andrea Cipollone, Simona Masina, Swadhin K. Behera

**Affiliations:** 1 Application Laboratory, VAiG, JAMSTEC, 3173-25, Showamachi, Kanazawa-ku, Yokohama City, Kanagawa 236-0001 Japan; 2 Ocean Modelling and Data Assimilation, CMCC, Bologna, Italy

**Keywords:** Physical oceanography, Cryospheric science

## Abstract

Skillful sea-ice prediction in the Antarctic Ocean remains a big challenge due to paucity of sea-ice observations and insufficient representation of sea-ice processes in climate models. Using a coupled general circulation model, this study demonstrates skillful prediction of the summertime sea-ice concentration (SIC) in the Weddell Sea with wintertime SIC and sea-ice thickness (SIT) initializations. During low sea-ice years of the Weddell Sea, negative SIT anomalies initialized in June retain the memory throughout austral winter owing to horizontal advection of the SIT anomalies. The SIT anomalies continue to develop in austral spring owing to more incoming solar radiation and the associated warming of mixed layer, contributing to further sea-ice decrease during late austral summer-early autumn. Concomitantly, the model reasonably reproduces atmospheric circulation anomalies during austral spring in the Amundsen-Bellingshausen Seas besides the Weddell Sea. These results provide evidence that the wintertime SIT initialization benefits skillful summertime sea-ice prediction in the Antarctic Seas.

## Introduction

Antarctic sea ice plays a key role in regional and global climate through changes in atmospheric and oceanic circulations over a wide range of spatiotemporal scales. Antarctic sea ice reaches its maximum extent in September and its minimum in March, and undergoes large interannual variations through remote atmospheric forcing and local air-sea-ice interaction. Most of the Antarctic Seas do not show any statistically significant trend in the sea-ice extent (SIE). The only exception is the increasing trend during the satellite observation period in the Ross Sea^1,2^. Efforts have been made to understand physical processes underlying interannual variability of the Antarctic sea ice. Both the atmospheric teleconnections from El Niño-Southern Oscillation (ENSO) and the Southern Annular Mode (SAM)^3,4^ are considered as key drivers for interannual sea-ice variations in the Antarctic Ocean through changes in regional atmospheric circulations^5,6^.

Using an atmospheric general circulation model, a previous study^7^ conducted sensitivity experiments and reported that recent sea-ice increase in the Ross and Amundsen-Bellingshausen Seas between 2000 and 2014 has a close link with a deepening of the Amundsen Sea Low through atmospheric teleconnection from the negative phase of the Interdecadal Pacific Oscillation (IPO)^8^. Furthermore, a positive phase of the SAM contributes to a negative trend of wind stress curl in the Southern Ocean, which enhances upwelling of warm water through Ekman suction and causes significant retreat of the Antarctic sea-ice after 2016^9^.

Atmospheric circulation changes associated with ENSO and the SAM are key sources of sea-ice predictability in the Antarctic Ocean, but the mechanisms behind these regional sea-ice predictabilities are not yet well established. In fact, a limited number of studies have reported low prediction skills of SIE in the Antarctic Ocean. For example, using global climate models, a previous study^10^ performed two reforecast experiments with/without sea-ice initialization and concluded that sea-ice initialization adds almost no values to prediction skills of the Antarctic SIE except during austral summer. The prediction skills of the Antarctic SIE are generally lower than the persistent skills using the observed sea-ice concentration (SIC) anomalies. Since the Antarctic SIC anomalies are spatially inhomogeneous with positive and negative signs, some parts of the Antarctic Ocean may show improvement of sea-ice prediction skills with sea-ice initialization. In fact, a long-term simulation of CGCM^11^ showed that there are regional differences in the sea-ice persistence predictability, with high predictability in the Indian Ocean sector and Amundsen-Bellingshausen Seas and low predictability in the Weddell and Ross Seas, respectively. Furthermore, a recent study^12^ has pointed out that the SIC initialization during austral winter using the observational data improves representation of the SIC anomalies in the Weddell Sea during austral spring, probably induced by local air-sea-ice interactions^13^. However, it remains a challenging issue to further extend the lead time of skillful sea-ice prediction beyond a season.

To tackle this unresolved issue, initialization of sea-ice thickness (SIT) using observational data or reanalysis product has received much attention as a potential way forward to performing the long-ranged sea-ice prediction. Using a regional ice-ocean model, the first attempt^14^ was made to correct the springtime SIT distribution with the observational data and to confirm that the model with the SIT initialization better estimates the summertime Arctic SIE than that without the SIT initialization. Using global climate and statistical models, subsequent studies^10,15–18^ have drawn similar conclusions, suggesting that the SIT initialization is crucial for extending a lead time of skillful sea-ice prediction in the Arctic Ocean, particularly during boreal summer. Recently, a few studies have made further attempts to utilize the CryoSat-2 satellite observation data for the SIT initialization^19–21^ and provided solid evidence for improvement of the Arctic sea-ice prediction during austral summer.

Given the above evidence in the Arctic Ocean, it is worth investigating the impact of SIT initialization on the prediction skills for the Antarctic sea ice. To achieve this objective, we conducted two reforecast experiments using a coupled general circulation model (CGCM) with/without the initialization of SIT from C-GLORS ocean reanalysis^22^ (see “Methods”). Comparison between the two experiments reveals to what degree the SIT initialization improves sea-ice prediction in the Antarctic Ocean, with a particular focus on the Weddell Sea where the SIT variability is pronounced, as discussed below. Also, we explore physical processes underlying the improvement of sea-ice prediction skills by conducting composite analysis for the low sea-ice years as an example in the Weddell Sea.

## Results

### Antarctic sea-ice climatology and variability

**Figure 1:**
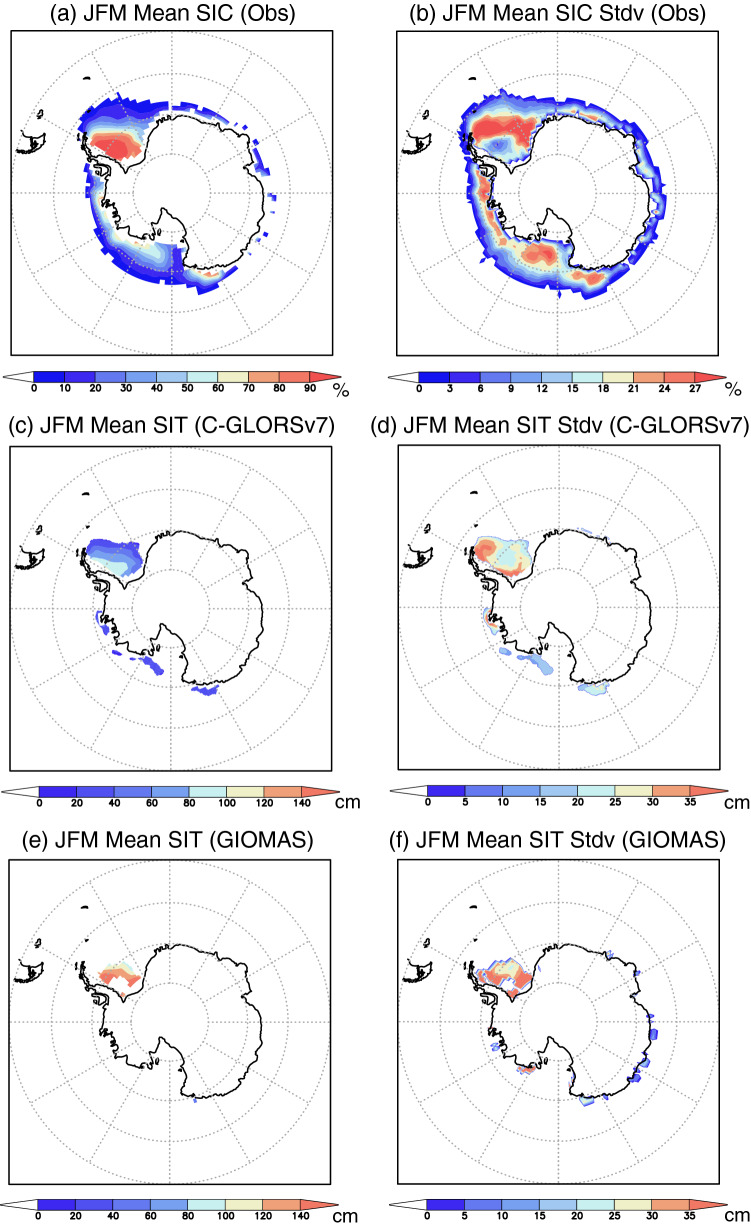
(**a**) Antarctic sea-ice concentration (SIC in %) during austral late summer-early autumn (January–March) from the OISSTv2 data. (**b**) Same as in (**a**), but for the standard deviation of the SIC (in %). (**c**) Antarctic sea-ice thickness (SIT in cm) during January–March from the C-GLORSv7 reanalysis. (**d**) Same as in (**c**), but for the standard deviation of the SIT (in cm). (**e**,**f**) Same as in (**c﻿**,**d**), but from the GIOMAS reanalysis.

Spatial pattern of the Antarctic sea ice shows a spatially inhomogeneous structure on seasonal-interannual timescales. Seasonal mean of the satellite SIC during late austral summer-early autumn (January–March) is large near the coasts of the Antarctica (Fig. 1a), with the largest meridional extent in the Weddell Sea. Standard deviation of the summertime SIC (Fig. 1b) is also large near the northern edge of the high SIC (Fig. 1a), and the SIC variability is meridionally the largest in the Weddell Sea. We obtain a similar tendency for the SIT mean state and its variability from two reanalysis products (C-GLORSv7^22^ and GIOMAS^23^; see “Methods”). Seasonal mean of the SIT during January–March shows high values in the Weddell Sea (Figs. 1c,e). Although there are some differences in the amplitude between the two reanalyses, spatial patterns are similar with the high SIT values confined to the western Weddell Sea. The standard deviation of the SIT during January–March shows a good agreement with the largest values in the western Weddell Sea (Figs. 1d,f).

In contrast, seasonal mean of the satellite SIC during austral winter (July–September) shows meridionally maximum extent in most of the Antarctic Ocean (Fig. S1a), with large standard deviation at the ice edge of the high SIC values (Fig. S1b). This indicates that air-sea-ice interactions take place actively to induce the SIC variability in the region with low SIC values. Seasonally averaged wintertime SIT from the two reanalyses has a spatially inhomogeneous structure with large values in the Weddell and Ross Seas (Figs. S1c, e). Also, the differences in the amplitude are large between the two reanalyses in contrast to that in late austral summer-early autumn (Fig. 1c,e). However, it is difficult to say which reanalysis is more realistic, because of lack of reliable observation data as a reference. For example, a previous study^24^ has discussed large uncertainties in the SIT observation data as there are large systematic and random measurement errors ($\pm$ 20–50%) by ship voyages or helicopter campaigns during 1981–2005. Also, other studies^25,26^ have claimed that there are large differences of the SIT measurements by up to 1 m between the Envisat and ICESat satellites in 2000s. Standard deviation of the SIT shows similarity with the largest values in the western Weddell Sea (Figs. S1d, f), where the SIC variability is small (Fig. S1b). Although there are some differences in the amplitude among the observational datasets, the C-GLORSv7 reanalysis has smaller absolute errors compared with those datasets and shows a good agreement with the multi-model ensemble mean SIT from the ten reanalysis products in terms of mean values and standard deviation^27^. Therefore, we have decided to use the C-GLORSv7 reanalysis to initialize the model’s SIT in this study.

### Sea-ice predictability in the Weddell Sea

**Figure 2:**
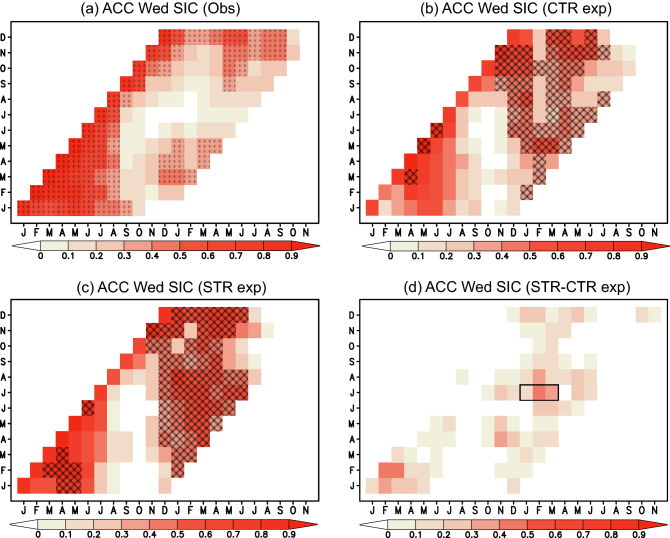
(**a**) Anomaly correlations (ACCs) for the persistence prediction using the observed SIC anomalies averaged in the Weddell Sea (70° S–60° S, 55° W–40° W; see the black boxes in Fig. 6). X-axis denotes predicted months, while Y-axis denotes initial months. Dots indicate correlations that are statistically significant at 90% confidence level using the two-tailed Student’s *t*-test. (**b**) Same as in (**a**), but for the ACCs between the observed SIC anomalies and the predicted SIC anomalies in the CTR experiment. Hatches indicate correlations above the persistence values that are statistically significant at 90% confidence level using the two-tailed Student’s *t*-test. (**c**) Same as in (**b**), but for the ACCs using the predicted SIC anomalies in the STR experiment. (**d**) Differences in the ACCs between the STR and CTR experiments, i.e., (**c**)–(**b**). Black box indicates January–March season of research interest.

Since the Weddell Sea shows the large seasonal-interannual sea ice variability in the Antarctic Ocean, we choose it to examine the impact of SIC and SIT initializations on sea-ice predictability using a CGCM (see “Methods”). Figure 2 shows monthly anomaly correlation (ACC) skills for the SIC anomalies averaged in the western Weddell Sea (70° S–60° S, 55° W–40° W) with the largest SIC and SIT variations. ACC skills (hereafter persistence skills) based on the observed SIC anomalies (Fig. 2a) are significantly high for wintertime SIC prediction during June–August, while the skills become low in other seasons, particularly during November-February. The ACC skills for the control (CTR) experiment with the SIC initialization (Fig. 2b; see “Methods”) are significantly higher than the persistence skills during December-May. We obtain a similar tendency for the sea-ice thickness restoring (STR) experiment with the SIC and SIT initializations (Fig. 2c; see “Methods”), but the differences in the ACC skills between the STR and CTR experiments show positive values during those seasons. In particular, the ACC differences are the largest with 0.31 on an average for the January–March mean SIC anomalies predicted from July 1st in the STR experiment (Fig. 2d; see the black box).

**Figure 3:**
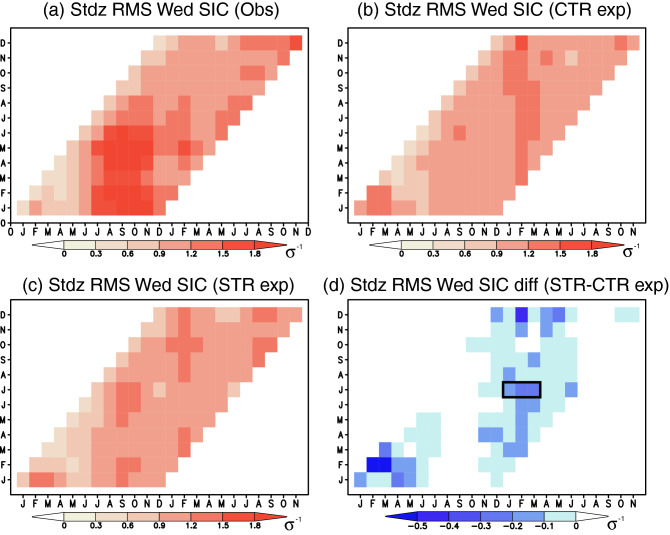
(**a**) Standardized root mean squared errors (RMSs) for the persistence prediction using the observed SIC anomalies averaged in the Weddell Sea (70° S–60° S, 55° W–40° W; see the black boxes in Fig. 6. X-axis denotes predicted months, while Y-axis denotes initial months. For standardization, we use the observed standard deviation for each month. (**b**) Same as in (**a**), but for the standardized RMSs between the observed SIC anomalies and the predicted SIC anomalies in the CTR experiment. (**c**) Same as in (**b**), but for the standardized RMSs using the predicted SIC anomalies in the STR experiment. (**d**) Differences in the standardized RMSs between the STR and CTR experiments, i.e., (**c**)–(**b**). Only negative values are shown to highlight improvement of the simulated amplitude in the STR experiment. A black box indicates January–March of research interest.

Standardized root mean squared errors (RMSs) for the SIC anomalies based on the observation (Fig. 3a) show large values during August-October when the sea ice reaches its maximum extent. Here we normalized RMSs with the observed standard deviation for each month, considering large differences in the monthly standard deviations. The standardized RMSs for the CTR experiment with the SIC initialization (Fig. 3b) are generally smaller than those for the observed SIC anomalies (Fig. 3a). We obtain a similar tendency for the STR experiment with the SIC and SIT initializations (Fig. 3c), but the differences in the standardized RMSs between the STR and CTR experiments (Fig. 3d) are mostly negative except during July-November. For example, the standardized RMSs for the STR experiment predicted from July 1st become smaller by about 0.2 standard deviation during January–March than those for the CTR experiment (Fig. S2). This makes the STR experiment skillful with the standardized RMSs lower than those for the persistence prediction. These statistical analyses indicate that the predicted amplitudes as well as signs of SIC anomalies are improved most effectively during January–March in the STR experiment.

**Figure 4:**
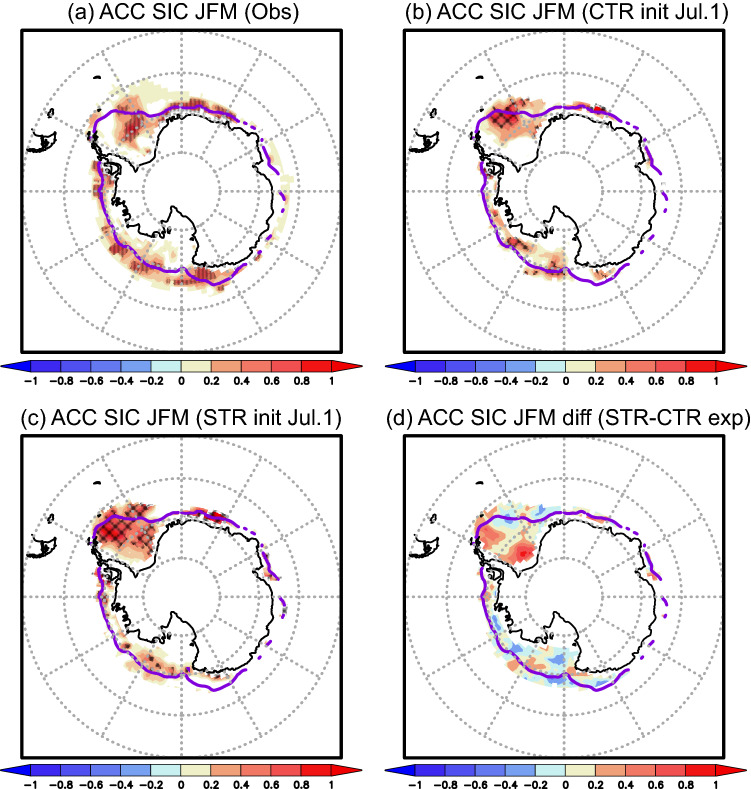
(**a**) ACCs between the observed SIC anomalies in June and the anomalies in January–March of the following year. Dots indicate correlations that are statistically significant at 90% confidence level using the two-tailed Student’s *t*-test. Purple lines represent sea-ice edges with the SIC values of 15% from the observation data. (**b**) Same as in (**a**), but for the January–March ACCs between the observed SIC anomalies and the SIC anomalies predicted from July 1st in the CTR experiment. Hatches indicate correlations above the persistence values that are statistically significant at 90% confidence level using the two-tailed Student’s *t*-test. (**c**) Same as in (**b**), but for the January–March ACCs between the observed SIC anomalies and the SIC anomalies predicted from July 1st in the STR experiment. (**d**) Differences in the January–March ACCs between the STR and CTR experiments, i.e., (**c**)–(**b**).

To provide a broad perspective on the impact of the wintertime SIT initialization on the summertime SIC prediction in the Antarctic Ocean, we discuss spatial patterns of the ACCs for the January–March mean SIC anomalies in the whole Antarctic Seas (Fig. 4). The persistence skills show moderately high values near the sea-ice edge, which are statistically significant, in the Weddell, Ross, and Amundsen-Bellingshausen Seas (Fig. 4a). The ACCs for the January–March mean SIC anomalies predicted from July 1st in the CTR experiment are slightly higher than the persistence skills in those seas (Fig. 4b), while the ACCs for the STR experiment are significantly higher, particularly in the Weddell Sea (Fig. 4c). The differences in the ACCs between the STR and CTR experiments show positive values in the western Weddell Sea (Fig. 4d), where the interannual SIT variability is large (Fig. 1d). Therefore, the Weddell Sea receives most benefits from the wintertime SIT initialization for skillful SIC prediction during late austral summer-early autumn, among the marginal seas of the Antarctic Ocean.

**Figure 5:**
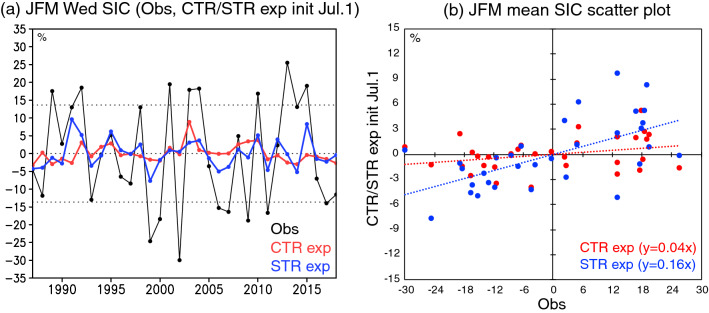
(**a**) Time series of January–March mean SIC anomalies (in % of total surface area) averaged in the Weddell Sea (70° S–60° S, 55° W–40° W; see the black boxes in Fig. 6). The observed anomalies (Obs; black line) and predicted anomalies from July 1st in the CTR (red line) and STR (blue line) experiments are shown, respectively. Dotted horizontal lines indicate positive and negative 0.9 standard deviations of the observed SIC anomalies. (**b**) Scatter plots of January–March mean SIC anomalies (in %) predicted from July 1st in the CTR (red dots) and STR (blue dots) experiments against the observed SIC anomalies averaged in the Weddell Sea. Dotted horizontal lines indicate linear regressions of the predicted anomalies.

To quantify the degree to which the prediction skills of the SIC anomalies are improved with the SIC and SIT initializations, we show time series of the SIC anomalies during January–March averaged in the western Weddell Sea (70° S–60° S, 55° W–40° W). This is a region where the large difference in the ACCs between the STR and CTR experiments is found (Fig. 5a). The correlation between the observed SIC anomalies and the SIC anomalies predicted from July 1st in the CTR experiment is low at 0.23, while that for the STR experiment is significantly high at 0.61. Also, the RMS between the observed SIC anomalies and the SIC anomalies predicted from July 1st in the CTR experiment is 15.0%, whereas that for the STR experiment becomes smaller at 13.3%. The RMS difference may be smaller than the uncertainties in the satellite SIC, but this reduction in the RMS makes the STR experiment skillful during January–March, as compared to the persistent prediction and the CTR experiment (Fig. S2). We obtain similar improvement of sea-ice prediction using scatter plots of the January–March mean SIC anomalies (Fig. 5b). A linear regression line of the predicted SIC anomalies against the observed anomalies during January–March has four-times gradient in the STR experiment (y = 0.16x) as large as that in the CTR experiment (y = 0.04x).

### Physical mechanisms on skillful sea-ice prediction in the Weddell Sea

**Figure 6:**
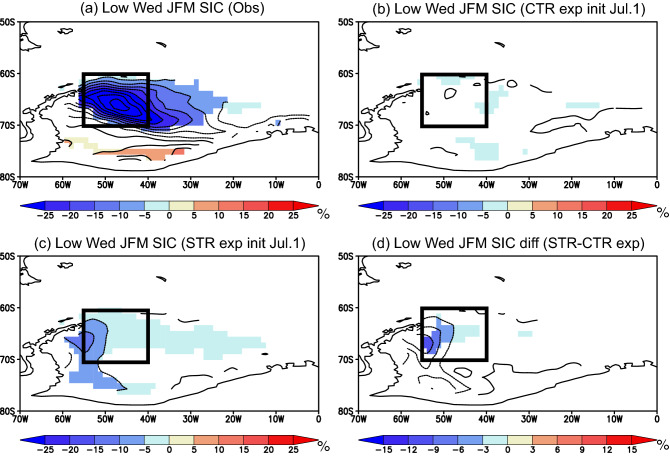
(**a**) Composite SIC anomalies (in %) observed during January–March of low sea-ice years in the Weddell Sea. Colors indicate anomalies that are statistically significant at 98% confidence level using the two-tailed Student’s *t*-test. A black box corresponds to the region of research interest (70° S–60° S, 55° W–40° W). (**b**) Same as in (**a**), but for the anomalies predicted from July 1st in the CTR experiment. (**c**) Same as in (**b**), but for the anomalies predicted from July 1st in the STR experiment. (**d**) Differences in the composite anomalies between the STR and CTR experiments, i.e., (**c**)–(**b**).

To explore the physical processes underlying the improved SIC predictions in the STR experiment, we conducted composite analysis for low sea-ice years in the Weddell Sea based on the time-series analysis of Fig. 5a (see “Methods” for definition of the low sea-ice years). It should be noted that composite results for the high sea-ice years have opposite signs, but the differences between the CTR and STR experiments are smaller and not significant (Fig. S3). Therefore, we discuss only the case for the low sea-ice years in this study. Composite SIC anomalies observed during January–March of low sea-ice years show large negative values with a northwest-southeast orientation in the Weddell Sea (Fig. 6a). The CTR experiment initialized on July 1st does not well reproduce the negative SIC anomalies in the Weddell Sea during January–March (Fig. 6b), while the STR experiment better captures the spatial pattern of the negative SIC anomalies (Fig. 6c). This agrees well with the statistical evidence that the pattern correlation of the SIC anomalies in the figure domain (80° S–50° S and 70° W–0° W) between the observation and the STR experiment is higher (0.40) than that for the CTR experiment (0.19). Although the STR experiment underestimates the amplitude of the observed SIC anomalies (Fig. 6a) as inferred from the time-series analysis in Fig. 5a, composite differences in the SIC anomalies between the CTR and STR experiments show significant improvement in the representation of the negative SIC anomalies in the western Weddell Sea (Fig. 6d; see the black box).

**Figure 7:**
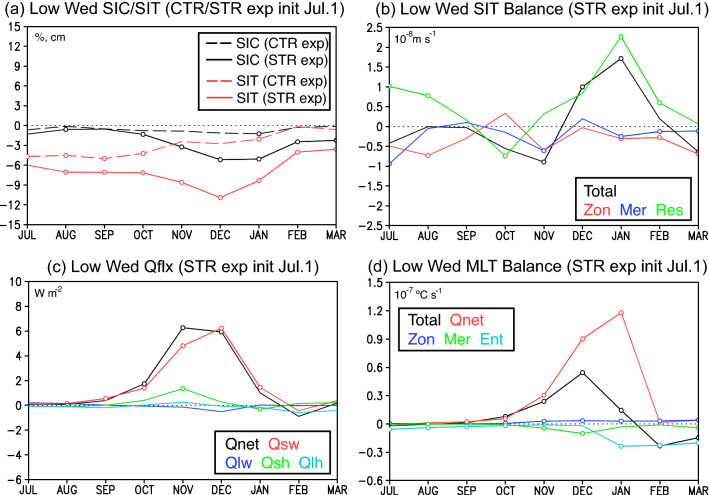
(**a**) Temporal evolution of composite SIC (black lines in %) and SIT (red lines in cm) anomalies in the Weddell Sea (70° S–60° S, 55° W–40° W; see the black boxes in Fig. 6) predicted from July 1st in the CTR (dashed lines) and STR (solid lines) experiments for low sea-ice years of the Weddell Sea. Open circles indicate anomalies that are statistically significant at 98% confidence level using the two-tailed Student’s *t*-test. (**b**) Same as in (**a**), but for composite anomalies of each term in the SIT balance equation (see Eq. (1) in “Methods”). The total SIT tendency (Total; black line in 10^–8^ m s^−1^), zonal (Zon; red line) and meridional (Mer; blue line) convergence/divergence, and residual (Res; green line) terms are shown, respectively. (**c**) Same as in (**a**), but for composite anomalies of surface heat fluxes onto the sea ice. Positive values indicate warming of the ocean. The net surface heat flux (Qnet; black line in W m^−2^), shortwave radiation (Qsw; red line), longwave radiation (Qlw; blue line), sensible heat flux (Qsh; green line), and latent heat flux (Qlh; light blue line) are shown, respectively. (**d**) Same as in (**a**), but for composite anomalies of each term in the mixed-layer temperature balance equation (see Eq. (2) in “Methods”). The mixed-layer temperature tendency (Total; black line in 10^–7^ °C s^−1^) and contributions from the net surface heat flux (Qnet; red line), zonal (Zon; blue line) and meridional (Mer; green line) advection, and entrainment (Ent; light blue line) terms are shown, respectively.

The differences in the SIC anomalies have a close link with those in the SIT anomalies. Time series of composite SIC anomalies averaged in the western Weddell Sea (70° S–60° S, 55° W–40° W) show almost no difference between the CTR and STR experiments during July–September (Fig. 7a), while those of composite SIT anomalies show a certain difference by a few cm during the period. The difference in the SIC and SIT anomalies between the two experiments becomes larger during October-December, but reduces during January–March. The reason for differences in the negative SIC anomalies predicted during January–March (Fig. 6d) is crucial to understand the temporal evolution of negative SIT anomalies before that season. For this purpose, we calculated composite anomalies of each term in the sea-ice thickness balance equation (see Eq. (1) in “Methods”) over the western Weddell Sea for the STR experiment (Fig. 7b). Tendency anomalies of the SIT are found to be negative from July to November. During July–August, the negative tendency anomalies are mostly due to contributions from zonal and meridional convergence of the negative SIT anomalies. Although the residual shows statistically significant positive anomalies, this acts to suppress the negative SIT tendency anomalies. These results indicate that the negative SIT anomalies initialized in the STR experiment can retain the memory owing to horizontal advection of the initial SIT anomalies. In October, the negative tendency anomalies of the SIT are also explained by contribution from the residual term that includes vertical processes, while in November, these are mostly due to zonal and meridional convergence of the negative SIT anomalies.

The vertical processes in the SIT balance involve both contributions from the overlying atmosphere and the ocean below sea ice. Time series of composite anomalies in the net surface heat flux and its component over the sea-ice/ocean (Fig. 7c) show that the net surface heat flux anomalies become significantly positive from October to December, mostly due to contribution from the positive shortwave radiation anomalies. This is explained by the decrease in the sea-ice cover as the incoming solar radiation increases owing to reduction of the surface albedo, performing as a positive feedback. Concomitantly, the mixed-layer temperature below the sea ice becomes significantly warmer than climatology from October (Fig. S4b). The warming of the mixed-layer is disconnected from the subsurface ocean which shows negative temperature anomalies below the mixed layer. The CTR experiment also shows warmer-than-normal temperature in the mixed layer (Fig. S4a), but the amplitude is much weaker than that in the STR experiment (Fig. S4b). This is evident in a remarkable difference of the subsurface ocean temperature anomalies in the mixed layer after November between the STR and CTR experiments (Fig. S4c).

Further analysis of mixed-layer temperature balance (see Eq. (2) in “Methods”) reveals that the tendency anomalies of the mixed-layer temperature become significantly positive since October (Fig. 7d). The positive tendency anomalies of the mixed-layer temperature are mostly explained by contribution from the surface heat flux term, in particular, shortwave radiation term (figure not shown). This indicates that as a result of sea-ice decrease, anomalous increase in the shortwave radiation contributes to warming of the mixed layer and this also influences sea-ice melting from the sea-ice bottom. Thus, both the incoming solar radiation and the associated warming of mixed layer provide favorable conditions for the growth of the negative SIT anomalies from October (Fig. 7b). In the STR experiment, the air-sea ice-mixed layer feedback process operates more effectively during austral spring when the SIC and SIT anomalies start to grow. The contributions of horizontal sea-ice transport and air-sea ice mixed-layer feedback process are found much weaker in the CTR experiment (Figs. S5a-c) due to the weaker amplitudes of the SIC and SIT anomalies (Fig. 7a).

**Figure 8:**
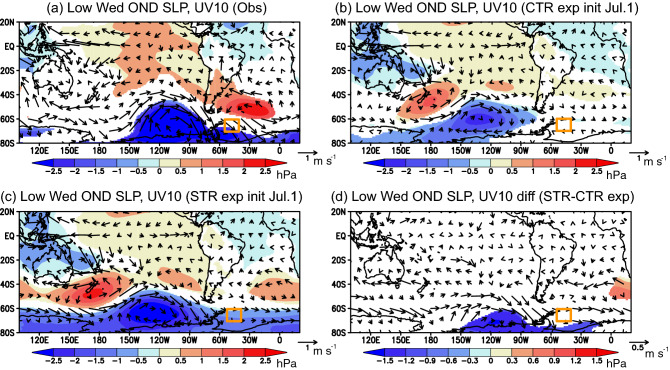
(**a**) Composite anomalies of sea-level pressure (SLP in hPa) and horizontal winds at 10 m from the surface (UV10 in m s^−1^) during October-December of low sea-ice years in the Weddell Sea. Atmospheric reanalysis is shown here. Colors indicate anomalies that are statistically significant at 98% confidence level using the two-tailed Student’s *t*-test. An orange box corresponds to the Weddell Sea region of interest. (**b**) Same as in (**a**), but for the anomalies predicted from July 1st in the CTR experiment. (**c**) Same as in (**b**), but for the anomalies predicted from July 1st in the STR experiment. (**d**) Differences in the composite anomalies between the STR and CTR experiments, i.e., (**c**)–(**b**).

SIT initialization improves the representation of the atmospheric circulation anomalies as well as sea-ice anomalies. During the growth phase of negative SIC anomalies (October-December), composite anomalies of SLP and horizontal winds at 10 m show that northeasterly wind anomalies associated with negative SLP anomalies prevail over the Weddell Sea (Fig. 8a). The negative SLP anomalies are partly linked to the negative surface air temperature (SAT) anomalies in the tropical Pacific representing La Niña conditions (Fig. S6a). This involves a wave train of positive and negative SLP anomalies in the South Pacific, like the Pacific-South American teleconnection (PSA)^28^. Also, the negative SLP anomalies in the Weddell Sea have a zonally elongated structure with positive SLP anomalies in the midlatitudes, representing a positive phase of the SAM. The CTR experiment does not well reproduce any of the negative SLP anomalies, the northwesterly wind anomalies, and positive SAT anomalies in the Weddell Sea (Figs. 8b, S6b). The STR experiment reasonably captures them in association with the positive phase of the SAM (Figs. 8c, S6c). The difference in the atmospheric circulation anomalies between the STR and CTR experiments shows significantly negative SLP anomalies in the Amundsen-Bellingshausen Seas besides the Weddell Sea (Fig. 8d). Since there are almost no significant differences in the SAT anomalies between the two experiments (Fig. S6d), the SIT initialization may improve representation of the SAM through modulation of the atmospheric internal variability at monthly or shorter timescales.

## Summary and discussion

This study has highlighted the importance of sea-ice thickness (SIT) initialization on skillful sea-ice prediction in the Antarctic Ocean, particularly in the Weddell Sea. The SIT anomalies initialized during austral winter using the C-GLORSv7 ocean reanalysis can be sustained via horizontal advection of the SIT anomalies and developed through air-sea ice-mixed layer feedback during austral spring. This leads to skillful prediction of the SIC anomalies during late austral summer-early autumn (January–March) with a lead time of 6–8 months. Compared to the SIC initialization that improves the spatial distribution of the SIC anomalies with a lead time of 1–3 months^12^, the SIT initialization extends the lead time of skillful sea-ice prediction across two seasons. Furthermore, the SIT initialization improves representation of atmospheric circulation anomalies in the Weddell and Amundsen-Bellingshausen Seas through modulation of atmospheric internal variability. Physical processes underlying the improvement of the atmospheric circulation anomalies remain unresolved and require further research using an atmospheric circulation model.

The SIT initialization adds positive values to sea-ice prediction skills not only in the Weddell Sea, as presented in the monthly ACC of SIC anomalies averaged in the pan-Antarctic Sea (Fig. S7). Persistent skills of the pan-Antarctic SIC anomalies based on the observation are very high (Fig. S7a), compared to the ACC skills obtained from the CTR and STR experiments (Fig. S7b, c). This was already pointed out using CGCMs^10^ with sea-ice initialization, but the differences in the ACC skills between the CTR and STR experiments show positive values added most effectively during December–April (Fig. S7d). This leads to the skillful prediction of the pan-Antarctic SIC anomalies in the STR experiment, in particular, during February–March (Fig. S7c). We obtain similar improvement for the standardized RMSs of the pan-Antarctic SIC anomalies (Fig. S8). Differences in the standardized RMSs between the STR and CTR experiments are generally negative during December–April (Fig. S8d), well consistent with the ACC differences (Fig. S7d). Although further efforts are needed to improve the pan-Antarctic sea-ice prediction, our study shows that the wintertime SIT initialization can benefit summertime sea-ice prediction even in the Antarctic Ocean.

A growing number of studies have reported significant improvement of the Arctic sea-ice prediction during boreal summer by using wintertime SIT initialization^10,15–21^ similar to what is shown in this study for the Antarctic Ocean. However, summertime SIT initialization is not yet seen to add positive values to wintertime sea-ice prediction skills in the Arctic^10^. We also find a similar issue on the lack of improvement of wintertime sea-ice prediction in the Antarctic Seas (Figs. 2, S2, S7, S8). The summertime SIT is so small in the very limited regions that the summertime SIT initialization does not have significant impacts on the wintertime sea-ice prediction. Rather, atmospheric and oceanic conditions may play important roles in determining wintertime sea-ice variability which is pronounced toward the mid-latitude edge of the sea-ice extent. Further initialization schemes such as inclusion of subsurface ocean temperature and salinity and an update freshwater input into the Antarctic Ocean^29^ would potentially improve the reproduction of sea-ice variability and its interplay with climate system. These would help skillful prediction of wintertime sea ice in the Antarctic Sea as well as the Arctic Sea^30^.

## Methods

### Observational data and reanalysis product

To compare with the results of model experiments, we analyzed monthly SST and SIC from the OISSTv2 dataset^31^ and SIT from the C-GLORSv7^22^ and GIOMAS^23^ reanalysis products, respectively. We also utilized atmospheric variables from the ERA-Interim reanalysis^32^. We obtained them with the same horizontal resolution of 1° × 1°, then smoothed them to fit into the horizontal resolution of the CGCM described below. We subtracted monthly climatology and linear trend over the analysis period of 1986–2017 to calculate monthly anomalies. To conduct composite analysis, we defined high and low sea-ice years in the Weddell Sea as years when the anomalies during January–March in the Weddell Sea (70° S–60° S, 55° W–40° W; see black boxes in Fig. 6) exceed positive and negative 0.9 standard deviations. This leads to 8 high sea-ice years (1989, 1992, 2001, 2003, 2004, 2010, 2013, 2015) and 8 low sea-ice years (1999, 2000, 2002, 2006, 2007, 2009, 2011, 2017), respectively. To test statistical significance for the correlation coefficients and composite anomalies, we adopted a two-tailed Student’s *t*-test with 90% and 98% confidence levels, respectively.

### CGCM reforecast experiments

To evaluate sea-ice prediction skills in the Antarctic Seas, we performed two reforecast experiments using a CGCM, called the Scale Interaction Experiment-Frontier Research Center for Global Change 2 (SINTEX-F2)^33–35^. This model reasonably simulates interannual sea-ice variability in the Weddell Sea^13^ and skillfully predicts the SIC during October-December when the model SIC is initialized with the observed SIC data on September 1st^12^. The atmospheric component of the SINTEX-F2 model is ECHAM5^36^ which has a T106 Gaussian grid with 31 vertical levels. The oceanic component of the SINTEX-F2 model is Nucleus for European Modeling of the Ocean (NEMO)^37^, which includes the Louvain-la-Neuve Sea Ice Model 2 **(**LIM2) sea ice model^38^ and has 0.5° × 0.5° horizontal resolution of ORCA tripolar grid (ORCA05) with 31 vertical levels. The SINTEX-F2 model exchanges the atmospheric and oceanic fields every two hours to compute air-sea momentum, heat and fresh water fluxes by means of the Ocean Atmosphere Sea Ice Soil 3 (OASIS3) coupler^39^.

We initialized the oceanic component of the SINTEX-F2 model using the mean temperature and salinity of World Ocean Database 1998, starting from the rest state^40^. Then, we spun up the SINTEX-F2 model with monthly climatology of the observed SST during 1950–1981. After that, we initialized the model SST with the observed SST every month during 1982–1985. To initialize the model SST, we nudged the model SST to the observed one by applying three negative feedback values (− 2400, − 1200, and − 800 W m^−2^ K^−1^) to the surface heat flux. For the initialization data, we used the weekly OISSTv2 dataset^31^ with a horizontal resolution of 1° × 1° and the daily OISSTv2 dataset^41^ with a horizontal resolution of 0.25° × 0.25°. Furthermore, we adopted two vertical mixing schemes to represent the impact of small vertical scale structures within and above the thermocline of the tropical oceans^34^. These differences in the initial conditions lead to generation of 12 ensemble members (Table S1). From 1986 to 2017, we conducted two reforecast experiments; in the CTR experiment, we initialized the model SST with the observed one from the OISSTv2 dataset and the model SIC with the one from the C-GLORSv7 reanalysis^22^, then integrated the model over 1 year starting from each month’s 1st of 1986–2017. In the STR experiment, we initialized the model SST with the observed one from the OISSTv2 dataset and the SIC and SIT with the ones from the ocean reanalysis, then integrated the model over 1 year for each month of 1986–2017. To initialize the model sea ice, we adopted a relaxation timescale of 5 days to nudge the model SIC and SIT to those from the ocean reanalysis. To calculate monthly anomalies, we subtracted monthly climatology and linear trend from the model output during the analysis period of 1986–2017.

### SIT and mixed-layer temperature balances

To explore the physical processes on the SIT variability, we calculated SIT tendency balance using a following equation:

1 $$\frac{\partial h}{{\partial t}} = - \frac{\partial hu}{{\partial x}} - \frac{\partial hv}{{\partial y}} + res,$$

where $h$ is the sea-ice thickness, $u$ and $v$ are the zonal and meridional sea-ice velocities, respectively. $res$ indicates vertical processes including both contributions from the overlying atmosphere and the ocean below sea ice. Here we calculated $res$ as a residual.

To investigate temporal evolution of mixed-layer temperature that impacts on sea-ice growth/decay from the sea-ice bottom, we also calculated a balance of mixed-layer temperature tendency using the following Eq. ^42^:

2 $$\frac{{\partial T_{m} }}{\partial t} = \frac{{Q_{net} - q_{d} }}{{\rho c_{p} H}} - u_{m} \frac{{\partial T_{m} }}{\partial x} - v_{m} \frac{{\partial T_{m} }}{\partial y} - \frac{{\Delta T}}{H}w_{e} + res,$$

where $T_{m}$ is the mixed-layer temperature, $Q_{net}$ is the net surface heat flux on the ocean surface, $q_{d}$ is the shortwave radiation that penetrates into the subsurface ocean below the mixed layer^43^, $\rho$ is the ocean density, $c_{p}$ is the ocean heat capacity, $H$ is the mixed-layer depth defined as the depth at which the potential ocean density increases by 0.03 kg m^−3^ compared to that at 10 m depth, $u_{m}$ and $v_{m}$ are the zonal and meridional velocities averaged in the mixed layer, $\Delta T$ is the differences in the mixed-layer temperature and the subsurface ocean temperature at the depth which is deeper by 20 m than the mixed-layer depth, and $w_{e} \left( { = \frac{\partial H}{{\partial t}} + \frac{{\partial Hu_{m} }}{\partial x} + \frac{{\partial Hv_{m} }}{\partial y}} \right)$ is the entrainment velocity. Here, $res$ includes vertical/horizontal diffusion and mixings and other unresolved processes.

## Supplementary Information

Supplementary Information.
