# Supplementary material for: Summertime sea-ice prediction in the Weddell Sea improved by sea-ice thickness initialization
Source: Sci Rep. 2021 Jun 9;11:11475. doi: 10.1038/s41598-021-91042-4 (PMC8190284; doi:10.1038/s41598-021-91042-4)
Supplement: Supplementary file 1 — Supplementary Information. [file 41598_2021_91042_MOESM1_ESM.docx]

**Supplementary information**

**“Summertime sea-ice prediction in the Weddell Sea improved by sea-ice thickness initialization”**

Yushi Morioka^1^, Doroteaciro Iovino^2^, Andrea Cipollone^2^,

Simona Masina^2^ and Swadhin K. Behera^1^

^1^*Application Laboratory, VAiG, JAMSTEC, Yokohama, Japan*

^2^*Ocean Modelling and Data Assimilation, CMCC, Bologna, Italy*

Corresponding author: Dr. Yushi Morioka

E-mail: [morioka@jamstec.go.jp](mailto:morioka@jamstec.go.jp)

**Supplementary Table and Figure Captions**

**Table S1:** Brief description of 12 ensemble members generated for the CTR and STR experiments. Here we used combination of three negative feedback values, two types of OISSTv2 datasets, and two types of vertical ocean mixing schemes to initialize 12 ensemble members.

F**igure S1:** Same as in Fig. 1, but for austral winter (July-September).

**Figure S2:** Standardized RMSs of the SIC anomalies averaged in the Weddell Sea (70ºS -60ºS, 55ºW-40ºW; see the black boxes in Fig. 6) for the persistence prediction (black line), CTR (red line) and STR (blue line) experiments initialized on July 1st.

**Figure S3:** (a) Composite SIC anomalies (in %) observed during January-March of high sea-ice years in the Weddell Sea. Colors indicate anomalies that are statistically significant at 98% confidence level using the two-tailed Student’s *t*-test. A black box corresponds to the region of research interest (70ºS -60ºS, 55ºW-40ºW). (b) Same as in (a), but for the anomalies predicted from July 1st in the CTR experiment. (c) Same as in (b), but for the anomalies predicted from July 1st in the STR experiment. (d) Differences in the composite anomalies between the STR and CTR experiments, i.e. (c)-(b).

**Figure S4:** (a) Temporal evolution of composite anomalies in the subsurface ocean temperature (in ºC) during low sea-ice years in the Weddell Sea. Predicted anomalies from July 1st in the CTR experiment are shown in color. Dots indicate anomalies that are statistically significant at 98% confidence level using the two-tailed Student’s *t*-test. A black line corresponds to the mixed-layer depth at which the potential ocean density increases by 0.03 kg m^-3^ compared to that at 10 m depth. (b) Same as in (a), but for the predicted anomalies from July 1st in the STR experiment. (c) Same as in (a), but for a difference in the subsurface ocean temperature anomalies between the STR and CTR experiments (i.e. (b)-(a)).

**Figure S5:** (a) Same as in Fig. 7b, but for composite anomalies of each term in the SIT balance equation (see Eq. (1) in Methods) for the CTR experiment. Open circles indicate anomalies that are statistically significant at 98% confidence level using the two-tailed Student’s *t*-test. The total SIT tendency (Total; black line in 10^-8^ m s^-1^), zonal (Zon; red line) and meridional (Mer; blue line) convergence/divergence, and residual (Res; green line) terms are shown, respectively. (b) Same as in Fig. 7c, but for composite anomalies of surface heat fluxes onto the sea-ice/ocean. Positive values indicate warming of the sea-ice/ocean. The net surface heat flux (Qnet; black line in W m^-2^), shortwave radiation (Qsw; red line), longwave radiation (Qlw; blue line), sensible heat flux (Qsh; green line), and latent heat flux (Qlh; light blue line) are shown, respectively. (c) Same as in Fig. 7d, but for composite anomalies of each term in the mixed-layer temperature balance equation (see Eq. (2) in Methods). The mixed-layer temperature tendency (Total; black line in 10^-7^ ºC s^-1^) and contributions from the net surface heat flux (Qnet; red line), zonal (Zon; blue line) and meridional (Mer; green line) advection, and entrainment (Ent; light blue line) terms are shown, respectively.

**Figure S6:** Same as in Fig. 8, but for composite anomalies of surface air-temperature (SAT in ºC) during October-December of the low sea-ice years in the Weddell Sea. Black boxes correspond to the Weddell Sea regions of interest.

**Figure S7:** Same as in Fig. 2, but for the ACCs using the SIC anomalies averaged in the Antarctic Ocean.

**Figure S8:** Same as in Fig. 3, but for the standardized RMSs using the SIC anomalies averaged in the Antarctic Ocean.

**Table S1:** Brief description of 12 ensemble members generated for the CTR and STR experiments. Here we used combination of three negative feedback values, two types of OISSTv2 datasets, and two types of vertical ocean mixing schemes to initialize 12 ensemble members.

| Negative feedback values | OISSTv2 dataset | Vertical ocean mixing |
| --- | --- | --- |
| -800 W m^-2^ K^-1^ | Daily (1986-2017) | With small vertical scale (SVS) mixing^34^ |
| -1200 W m^-2^ K^-1^ | Weekly (1986-2017) | Without SVS mixing^34^ |
| -2400 W m^-2^ K^-1^ |  | |


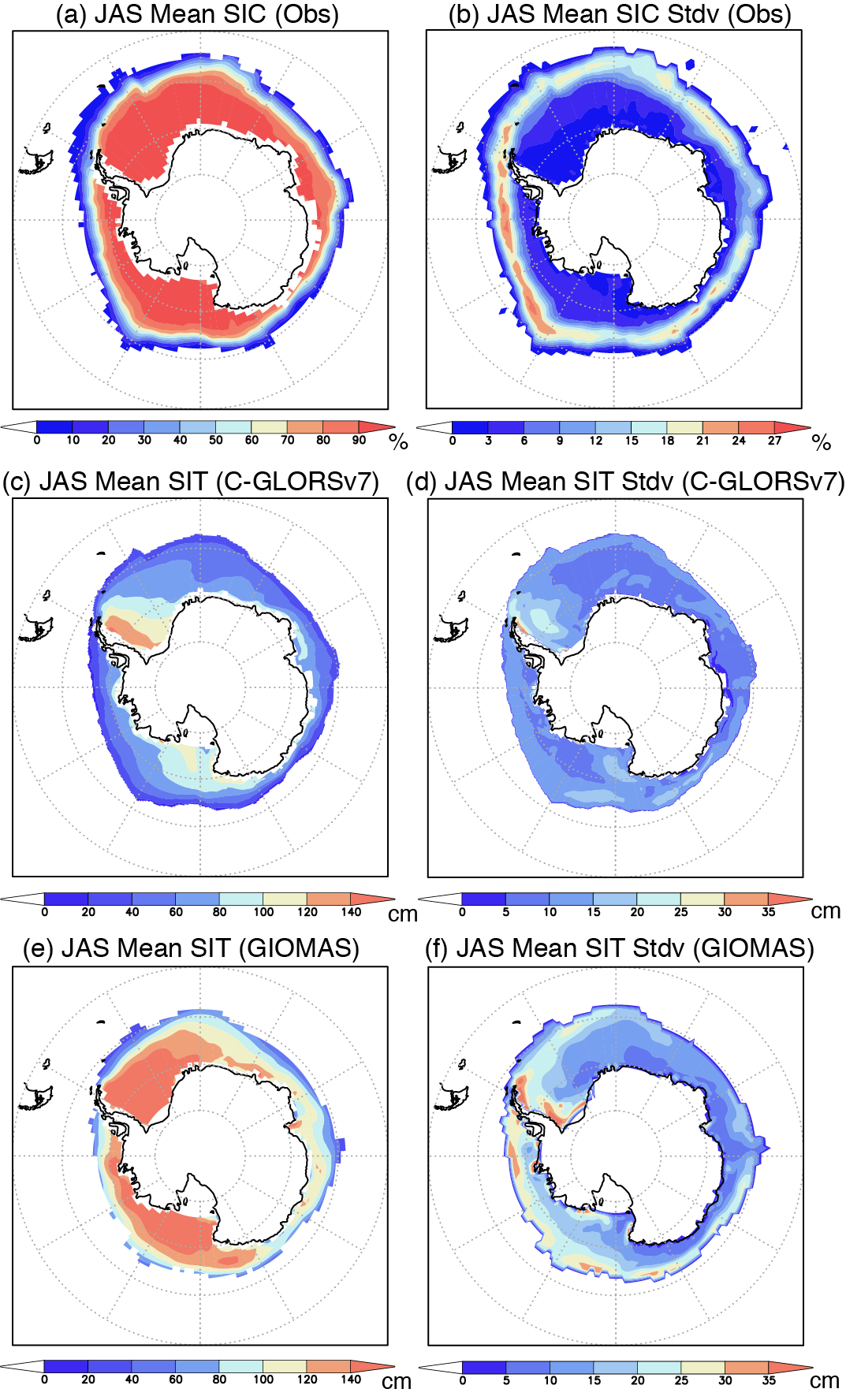


F**igure S1:** Same as in Fig. 1, but for austral winter (July-September).

**
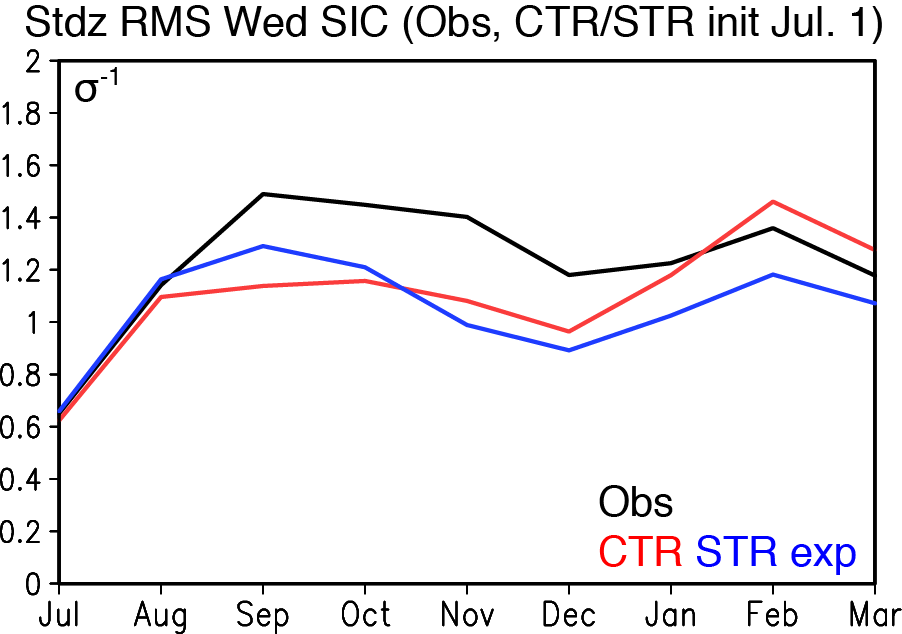
**

**Figure S2:** Standardized RMSs of the SIC anomalies averaged in the Weddell Sea (70ºS -60ºS, 55ºW-40ºW; see the black boxes in Fig. 6) for the persistence prediction (black line), CTR (red line) and STR (blue line) experiments initialized on July 1st.

**
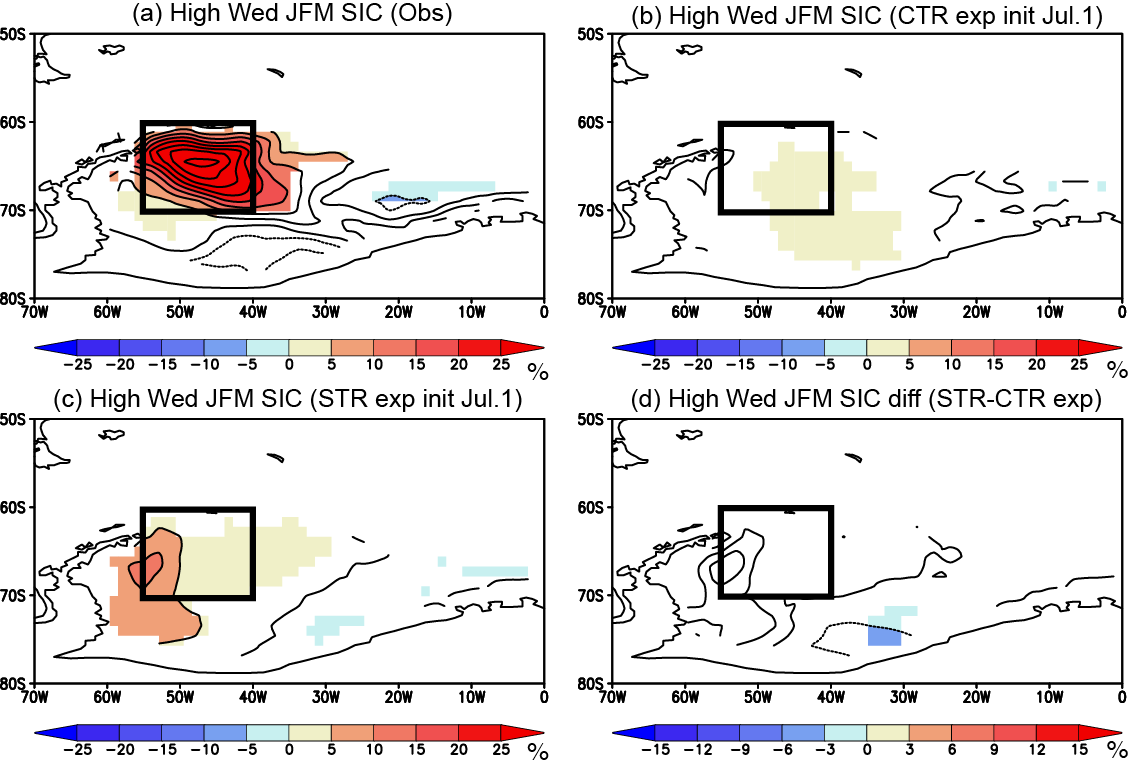
**

**Figure S3:** (a) Composite SIC anomalies (in %) observed during January-March of high sea-ice years in the Weddell Sea. Colors indicate anomalies that are statistically significant at 98% confidence level using the two-tailed Student’s *t*-test. A black box corresponds to the region of research interest (70ºS -60ºS, 55ºW-40ºW). (b) Same as in (a), but for the anomalies predicted from July 1st in the CTR experiment. (c) Same as in (b), but for the anomalies predicted from July 1st in the STR experiment. (d) Differences in the composite anomalies between the STR and CTR experiments, i.e. (c)-(b).


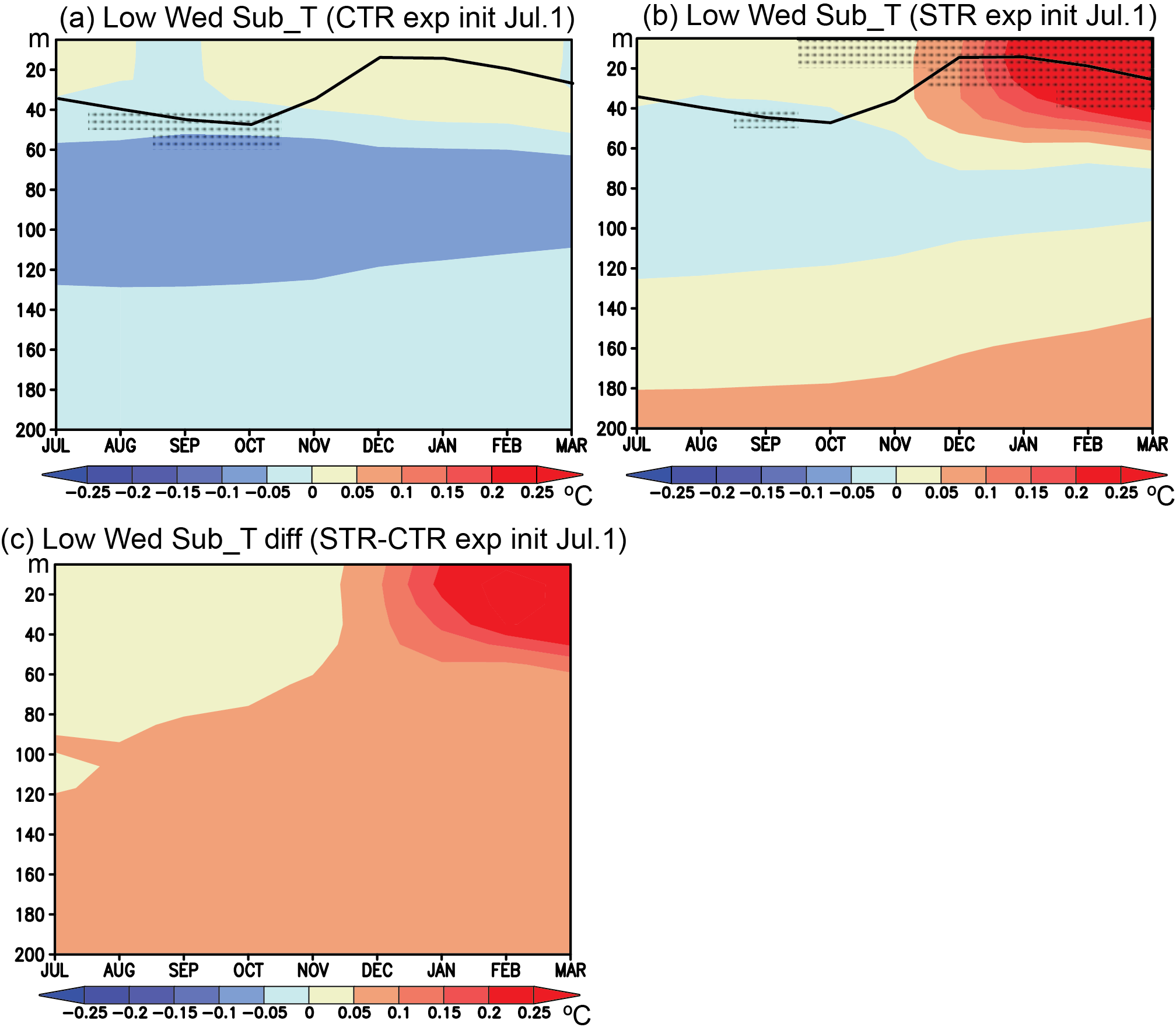


**Figure S4:** (a) Temporal evolution of composite anomalies in the subsurface ocean temperature (in ºC) during low sea-ice years in the Weddell Sea. Predicted anomalies from July 1st in the CTR experiment are shown in color. Dots indicate anomalies that are statistically significant at 98% confidence level using the two-tailed Student’s *t*-test. A black line corresponds to the mixed-layer depth at which the potential ocean density increases by 0.03 kg m^-3^ compared to that at 10 m depth. (b) Same as in (a), but for the predicted anomalies from July 1st in the STR experiment. (c) Same as in (a), but for a difference in the subsurface ocean temperature anomalies between the STR and CTR experiments (i.e. (b)-(a)).

**
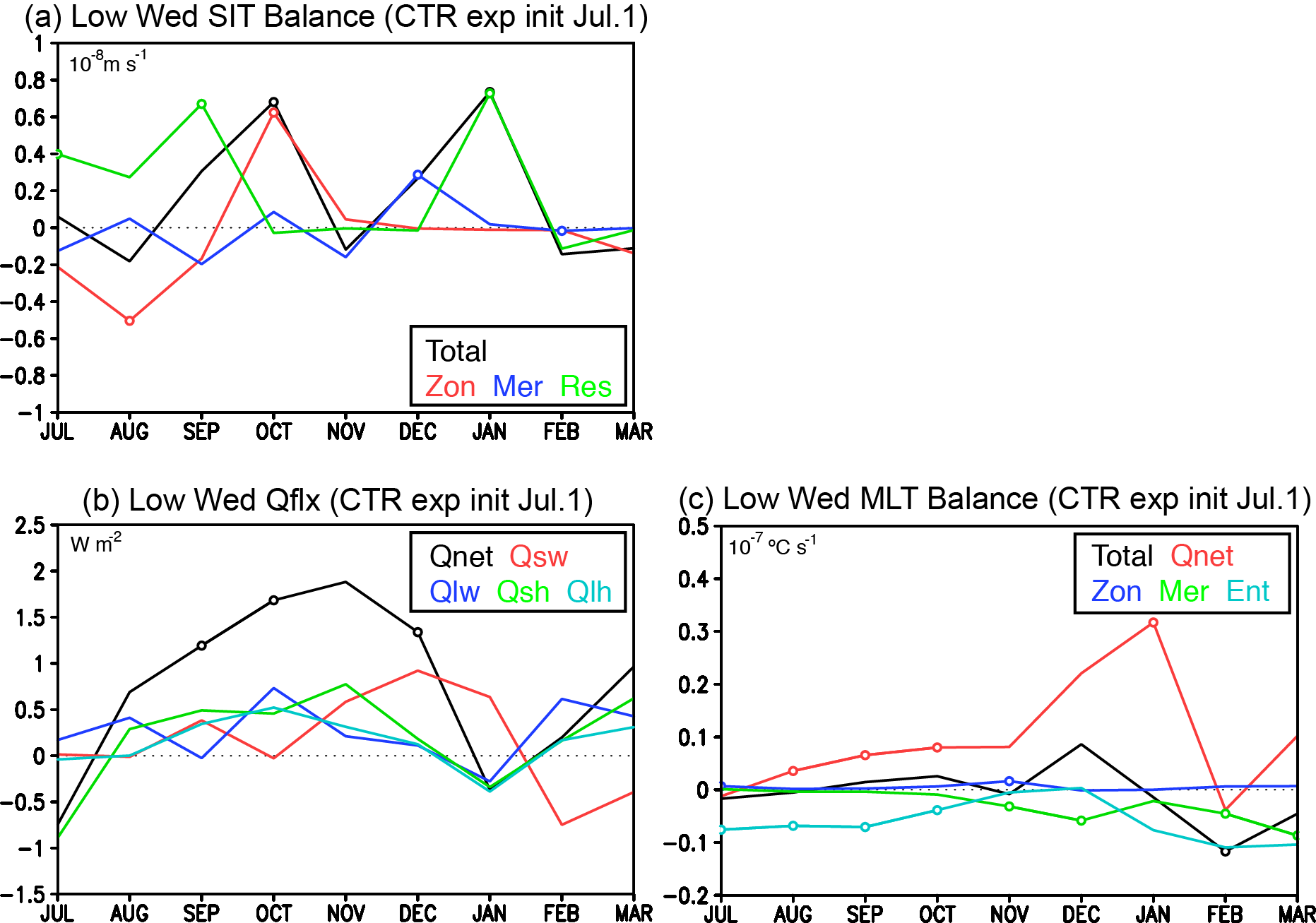
**

**Figure S5:** (a) Same as in Fig. 7b, but for composite anomalies of each term in the SIT balance equation (see Eq. (1) in Methods) for the CTR experiment. Open circles indicate anomalies that are statistically significant at 98% confidence level using the two-tailed Student’s *t*-test. The total SIT tendency (Total; black line in 10^-8^ m s^-1^), zonal (Zon; red line) and meridional (Mer; blue line) convergence/divergence, and residual (Res; green line) terms are shown, respectively. (b) Same as in Fig. 7c, but for composite anomalies of surface heat fluxes onto the sea-ice/ocean. Positive values indicate warming of the sea-ice/ocean. The net surface heat flux (Qnet; black line in W m^-2^), shortwave radiation (Qsw; red line), longwave radiation (Qlw; blue line), sensible heat flux (Qsh; green line), and latent heat flux (Qlh; light blue line) are shown, respectively. (c) Same as in Fig. 7d, but for composite anomalies of each term in the mixed-layer temperature balance equation (see Eq. (2) in Methods). The mixed-layer temperature tendency (Total; black line in 10^-7^ ºC s^-1^) and contributions from the net surface heat flux (Qnet; red line), zonal (Zon; blue line) and meridional (Mer; green line) advection, and entrainment (Ent; light blue line) terms are shown, respectively.


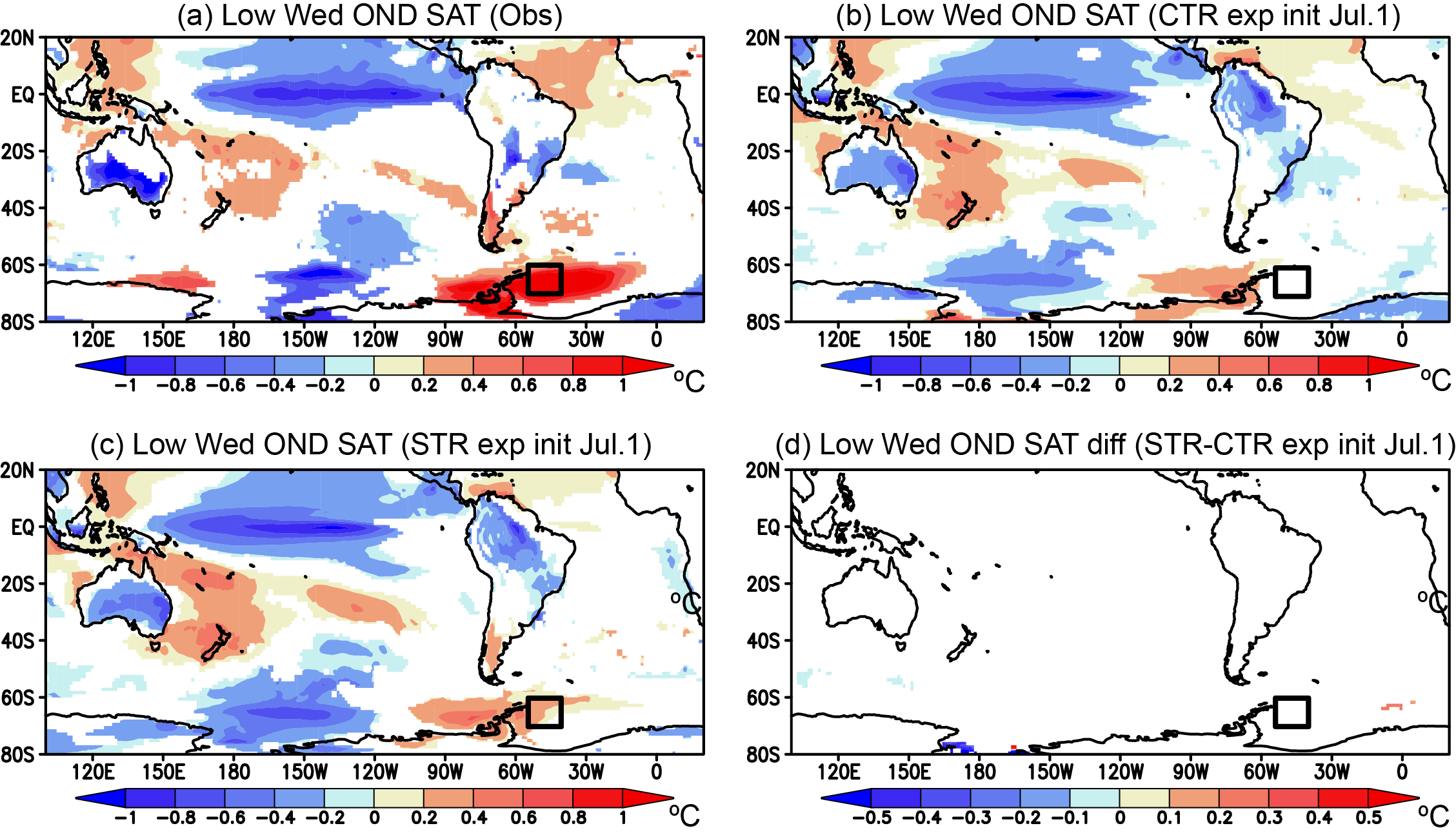


**Figure S6:** Same as in Fig. 8, but for composite anomalies of surface air-temperature (SAT in ºC) during October-December of the low sea-ice years in the Weddell Sea. Black boxes correspond to the Weddell Sea regions of interest.


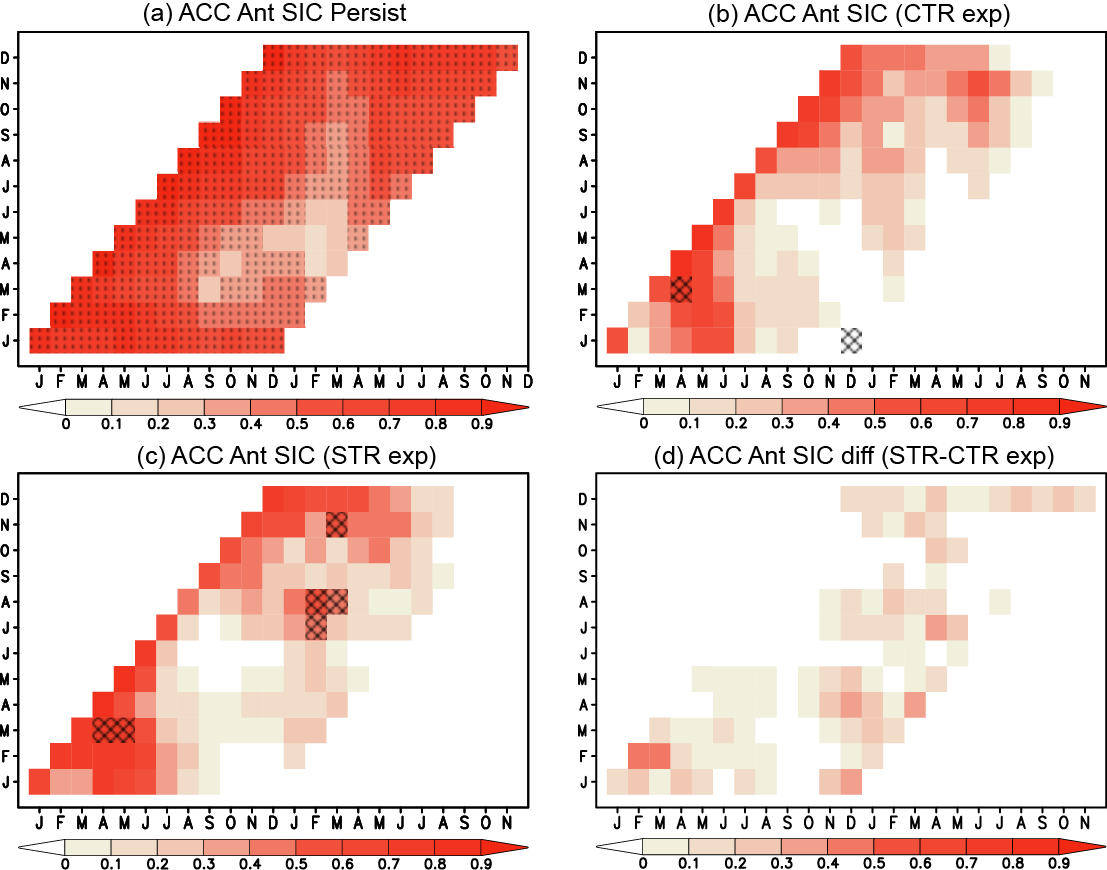


**Figure S7:** Same as in Fig. 2, but for the ACCs using the SIC anomalies averaged in the Antarctic Ocean.

**
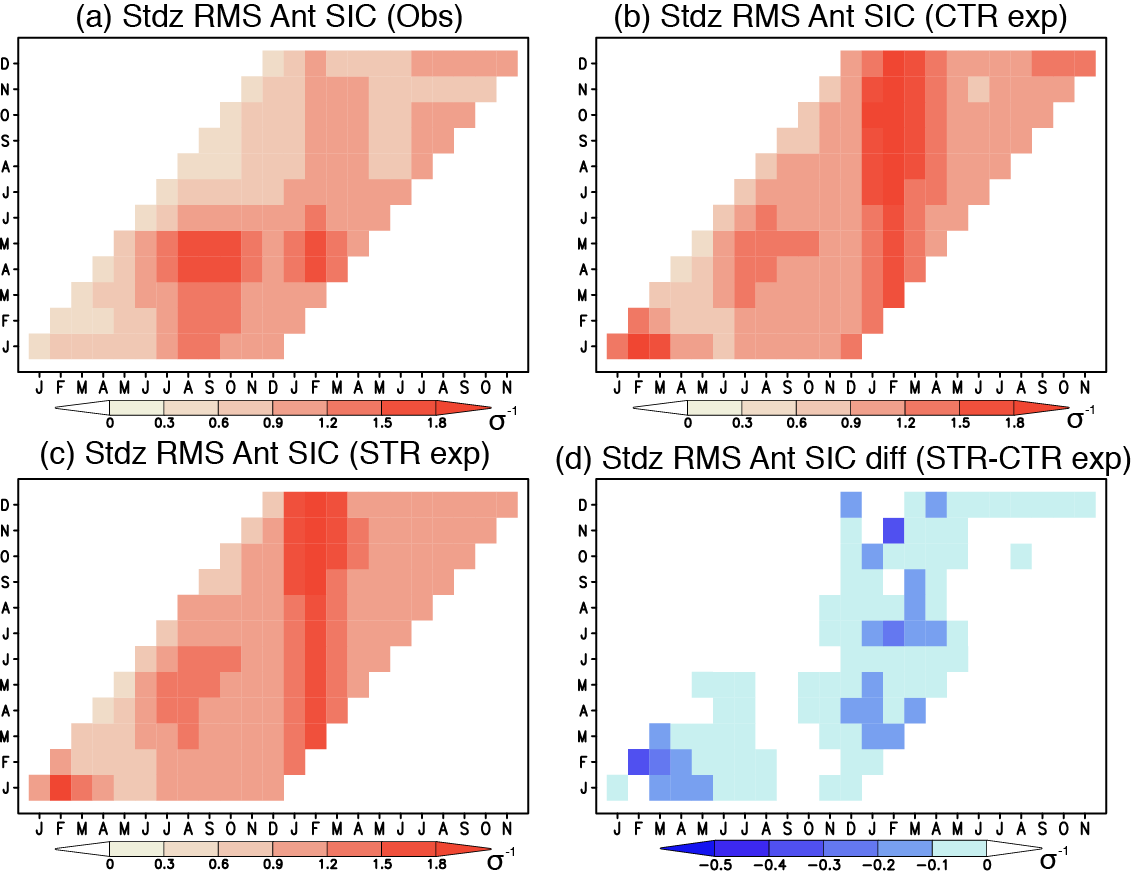
**

**Figure S8:** Same as in Fig. 3, but for the standardized RMSs using the SIC anomalies averaged in the Antarctic Ocean.
